# Initial Solution Generation and Diversified Variable Picking in Local Search for (Weighted) Partial MaxSAT

**DOI:** 10.3390/e24121846

**Published:** 2022-12-18

**Authors:** Zaijun Zhang, Jincheng Zhou, Xiaoxia Wang, Heng Yang, Yi Fan

**Affiliations:** 1 School of Mathematics and Statistics, Qiannan Normal University for Nationalities, Duyun 558000, China; 2 Key Laboratory of Complex Systems and Intelligent Optimization of Guizhou Province, Duyun 558000, China; 3 School of Computer and Information, Qiannan Normal University for Nationalities, Duyun 558000, China; 4 Guangxi Key Laboratory of Trusted Software, Guilin University of Electronic Technology, Guilin 541004, China

**Keywords:** maximum satisfiability, structural entropy, local search, heuristic search

## Abstract

The (weighted) partial maximum satisfiability ((W)PMS) problem is an important generalization of the classic problem of propositional (Boolean) satisfiability with a wide range of real-world applications. In this paper, we propose an initialization and a diversification strategy to improve local search for the (W)PMS problem. Our initialization strategy is based on a novel definition of variables’ structural entropy, and it aims to generate a solution that is close to a high-quality feasible one. Then, our diversification strategy picks a variable in two possible ways, depending on a parameter: continuing to pick variables with the best benefits or focusing on a clause with the greatest penalty and then selecting variables probabilistically. Based on these strategies, we developed a local search solver dubbed ImSATLike, as well as a hybrid solver ImSATLike-TT, and experimental results on (weighted) partial MaxSAT instances in recent MaxSAT Evaluations show that they outperform or have nearly the same performances as state-of-the-art local search and hybrid competitors, respectively, in general. Furthermore, we carried out experiments to confirm the individual impacts of each proposed strategy.

## 1. Introduction

The maximum satisfiability (MaxSAT) problem is an optimization version of the Boolean satisfiability (SAT) problem, which is a prototypical NP-complete problem. In the context of the SAT and MaxSAT problems, a propositional formula *F* is usually expressed in conjunctive normal form (CNF) [1], i.e., $F={⋀}_{i}{⋁}_{j}l_{ij}$, where each $l_{ij}$ is a literal, which is either a Boolean variable or its negation. A CNF formula can be expressed as a set of clauses, where a clause is a disjunction of literals, and each CNF formula is a conjunction of clauses.

Given a formula in CNF, the MaxSAT problem is to seek an assignment that minimizes the number of unsatisfied clauses in the formula. The partial maximum satisfiability (PMS) problem generalizes the MaxSAT problem to involve both hard and soft clauses. It aims to find a solution that minimizes the number of violated soft clauses while satisfying all the hard ones. The weighted partial maximum satisfiability (WPMS) problem is a generalization of the PMS problem, which further associates each soft clause with a positive weight and tries to locate a solution that minimizes the total weight of violated soft clauses. The MaxSAT, PMS, and WPMS problems are all NP-hard, and it is well known that optimum solutions are hard to approximate [2]. Obviously, MaxSAT is a special case of PMS, where the set of hard clauses is empty, and PMS is a special case of WPMS, where each soft clause is associated with the same weight.

Like other combinatorial problems, real-world applications usually contain hard and soft constraints [3], and soft ones often have different importance. Encoding such problems into PMS and WPMS problems is natural and straightforward [4,5,6,7]. In fact, real-world problems such as computational protein design [8,9], set covering [10], coalition structure generation [11], and large-scale road sensing through crowdsourced vehicles [12] can be encoded and solved as PMS or WPMS problems.

There are two popular kinds of algorithms for solving MaxSAT and also its extensions: complete and stochastic local search (SLS) algorithms. Complete algorithms are able to confirm the optimality of the returned solution at the end, but they may fail to return a high-quality one for large-scale instances within reasonable time [13]. These algorithms can further be classified into two main subcategories: branch and bound MaxSAT algorithms [14,15,16,17], which are based on David–Putnam–Loveland–Logemann (DPLL) procedures [18,19], and SAT-based ones [20,21,22,23,24,25,26,27], which call efficient conflict-driven clause learning (CDCL) SAT solvers [28,29] to solve a sequence of SAT problems. Considering recent MaxSAT Evaluations, we found that branch and bound algorithms are superior on crafted benchmarks, while SAT-based ones perform better on application benchmarks (https://maxsat-evaluations.github.io/2018/, accessed on 8 November 2022). Furthermore, SAT-based solvers, namely Open-WBO [30], LinSBPS, and TT-Open-WBO-inc [31], performed extraordinarily in incomplete solver tracks of MaxSAT Evaluations 2018 and 2019 (https://helda.helsinki.fi/bitstream/handle/10138/237139/mse18_proceedings.pdf?sequence=1, https://helda.helsinki.fi/bitstream/handle/10138/306989/mse19proc.pdf?sequence=1 accessed on 8 November 2022). On the other hand, SLS algorithms are often able to find satisfactory solutions within a reasonable time frame [3,32], although they do not guarantee the optimality of the solution they find. These algorithms are usually variants or refinements of two prototype solvers, i.e., GSAT [33] and WalkSAT [34].

### 1.1. Local Search for MaxSAT

Recently, significant breakthroughs have been achieved by SLS algorithms for solving PMS and WPMS problems, resulting in state-of-the-art SLS algorithms, namely Dist [3] together with its improvement DistUP [35], CCEHC [36], and SATLike together with one of its variants SATLike 3.0 [37]. The Dist algorithm shows great success in solving PMS and won several categories in the incomplete solver track of the MaxSAT Evaluation 2014. Furthermore, it competes well with state-of-the-art complete algorithms on some classes of PMS application instances, such as advanced encryption standard and protein [3]. Furthermore, Dist can also be adapted to solve WPMS and is still one of the current best SLS algorithms for solving WPMS. The DistUP algorithm, an improvement of Dist, which incorporates unit propagation in its initialization procedure, shows improvement over Dist on industrial instances. However, CCLS, Dist, and DistUP are not dedicated to solving WPMS, and their performance for solving WPMS could be further improved. This motivates the design of a solver dubbed CCEHC [36], which is the state-of-the-art on WPMS instances. The CCEHC algorithm extends the framework of CCLS with an extra heuristic, which emphasizes hard clauses (EHCs). With a strong focus on hard clauses, the EHC heuristic has three components: a variable selection mechanism, which focuses on a forbidding mechanism called configuration checking based only on hard clauses, a weighting scheme for hard clauses, as well as an approach of a biased random walk. Later, SATLike and its variant SATLike 3.0 outperformed previous solvers in solving PMS and WPMS problems. Moreover, they are thought to be the first SLS solvers that compete well with SAT-based ones.

Despite the significant breakthroughs above, there is still a gap between the performances of SLS solvers and those of SAT-based ones. To make matters worse, the algorithms for the former ones are more complicated than those for the latter ones. We believe that these drawbacks may be due to certain structures of PMS and WPMS problems. For example, there are two kinds of clauses, hard and soft ones. Furthermore, these drawbacks could also be caused by improper selections of initial solutions (starting points of local search) or diversifying variables. In this sense, the detailed analysis of the structures of PMS and WPMS problems, as well as suitable initial solutions and diversifying variables may lead to significant improvements.

### 1.2. Our Contributions

In this work, we develop an SLS solver named ImSATLike together with a hybrid one dubbed ImSATLike-TT based on two novel strategies, i.e., generating a high-quality starting point for local search and selecting a promising variable for diversification. Firstly, our initial solution generation is based on a notion called *variable entropy*. The resulting solution is closer to high-quality feasible solutions compared to those generated in the most common and traditional approach, i.e., pure random assignments. Experiments showed that this strategy is able to improve the efficiency of locating a satisfactory solution. Secondly, when the search is trapped in local optima, it will focus more on three types of variables: (1) those in the whole formula, which has the greatest benefit; (2) those lying in a clause with the greatest penalty; (3) those causing the least clauses to become unsatisfied. Thirdly, we also develop a hybrid solver ImSATLike-TT, which combines ImSATLike with a state-of-the-art SAT-based solver TT-Open-WBO-inc [31], and this solver presents satisfactory performances on (weighted) partial MaxSAT instances in recent MaxSAT Evaluations.

The rest of this paper is organized as follows. Some necessary concepts and basic notations are introduced in Section 2. The strategy of generating an initial solution based on variables’ structural entropy is introduced in Section 3. In Section 4, we introduce the diversifying variable selection strategy based on clause penalties. Our algorithm and the experimental evaluations are presented in Section 5 and Section 6, respectively. In Section 7, we give some conclusions and the future work.

## 2. Preliminaries

Throughout this paper, we talk about propositional logic. Given a set of *n* Boolean variables (also called propositional atoms) $V=\{x_{1},\cdots ,x_{n}\}$, a literal *l* is either $x_{k}$ or $\neg x_{k}$, where k=1,2,⋯,n. A clause $C=l_{1}\vee \cdots \vee l_{s}$ is a disjunction of literals, where *s* is called the (clause) length of *C*. Then, we use V(C) as the set of variables in *C*. In addition, if $l=x_{k}$ (respectively $l=\neg x_{k}$) is a literal in *C*, then we say that $x_{k}$ occurs positively (respectively negatively) in *C*, and we can also say that *C* contains $x_{k}$’s positive (respectively negative) occurrence.

A formula *F* in conjunctive normal form (CNF) is a conjunction of clauses, i.e., $F=C_{1}\wedge \cdots \wedge C_{t}$, where *t* is called the number of clauses in *F*. Given a CNF formula *F*, we abuse V(F) to denote the set of variables in *F*, i.e., $V\left(F\right)={⋃}_{1\leq j\leq t}V\left(C_{j}\right)$. Furthermore, we use C(F) to denote the set of clauses in *F*, i.e., $C\left(F\right)=\left\{C_{1},\cdots ,C_{t}\right\}$. In the MaxSAT problem, as well as its variants, clauses are usually partitioned into hard and soft ones, so we use $C_{h}\left(F\right)$ and $C_{s}\left(F\right)$ to denote the set of hard and soft clauses in *F*, respectively.

Two different variables, namely *x* and *y*, are said to be neighbors if there exists at least one clause *C* in C(F) s.t. both *x* and *y* occurring in *C*. We use N(x,F) to denote the set of *x*’s neighboring variables in *F*, i.e., N(x,F)={y|x,ybothoccurinC,C∈C(F)}. Given a CNF formula *F* with a weighting function $W_{F}:C\left(F\right)\mapsto Z^{+}$, we say that $\langle F,W_{F}\rangle$ is a MaxSAT formula (or we call it a MaxSAT instance). Without loss of generality for any unweighted soft clause $C^{s}\in C\left(F\right)$, we let $W_{F}\left(C^{s}\right)=1$. We use $W_{F}$ with subscripts here in order to distinguish between this weight notation and those below in graph theory.

Usually, SLS algorithms will first make a random guess to obtain a candidate solution, then they will change this solution by trial and error, so we introduce some related notions here. A complete assignment is a map α:V(F)↦{0,1}, which assigns a Boolean value (either 0 or 1) to each variable in the formula *F*, so for any variable *x* in *F*, either α(x)=0 or α(x)=1. In the context of SLS algorithms for MaxSAT, as well as its variants, a (candidate) solution is a complete assignment. In this sense, we say that *x* is flipped if we change the Boolean value of *x* from 0 to 1 or vice versa. More formally, this manipulation leads to another assignment $\alpha^{\prime }=\alpha \backslash \left\{\langle x,\alpha \left(x\right)\rangle \right\}\cup \left\{\langle x,1-\alpha \left(x\right)\rangle \right\}$. In what follows, we will use the notions of assignment and solution interchangeably.

Given a CNF formula *F* and a complete assignment α that maps V(F) to {0,1}, each clause in *F* under the assignment α has two possible states: satisfied and unsatisfied; a clause *C* in *F* is satisfied if at least one literal in *C* takes the value 1 (*true*) under α; otherwise, *C* is unsatisfied.

Clauses in a (weighted) partial MaxSAT formula $\langle F,W_{F}\rangle$ are partitioned into hard and soft ones, and each soft clause in the weighted case is further associated with a positive integer. Given $\langle F,W_{F}\rangle$, the (weighted) partial maximum satisfiability ((W)PMS)) problem is to find a complete assignment that satisfies all hard clauses in *F* and minimizes the total weight/number of all unsatisfied soft clauses in *F*.

Given a (weighted) partial MaxSAT formula $\langle F,W_{F}\rangle$, a complete assignment is feasible if it satisfies all hard clauses in *F*. The quality of a complete assignment α over $\langle F,W_{F}\rangle$, denoted as $\mathrm{quality}(\alpha ,F,W_{F})$, is the total weight/number of all satisfied soft clauses in $\langle F,W_{F}\rangle$ under α. An optimum assignment is a feasible assignment, namely $\alpha^{*}$, s.t., for any feasible assignment α over $\langle F,W_{F}\rangle$, $\mathrm{quality}\left(\alpha^{*},F,W_{F}\right)\geq \mathrm{quality}\left(\alpha ,F,W_{F}\right)$, that is an optimum assignment over $\langle F,W_{F}\rangle$ is an feasible assignment with the minimum cost. In what follows, we usually suppress *F* and $W_{F}$ in $\mathrm{quality}(\alpha ,F,W_{F})$ if understood from the context.

### 2.1. Variable Graph

The research community for complex networks has developed techniques of analysis and algorithms to study real-world graphs, and such approaches can be adopted by the SAT community. Inspired by the results on complex networks, Ref. [21] studied the community structure, or modularity, of industrial SAT instances, and they proposed a notion named the *variable graph*, which describes the interactions between Boolean variables in a SAT formula. Here, we extend the notion of the *variable graph* so that it works seamlessly in PMS and WPMS problems.

The variable graph of a (weighted) partial MaxSAT formula $\langle F,W_{F}\rangle$, denoted by $G(F,W_{F})$, is defined as $\left(V_{F},E_{F},W_{\langle F,W_{F}\rangle }\right)$, which describes the interactions between any pair of distinct Boolean variables in *F*. First, $V_{F}$ is a vertex set s.t. each vertex $v_{i}\in V_{F}$ representing a Boolean variable $x_{i}\in V\left(F\right)$, i.e., there is a bijection $\phi :V\left(F\right)\mapsto V_{F}$ for graph construction. In this sense, the inverse function $\phi^{-1}$ exists, and it maps vertices, namely $v_{i}$, back to their corresponding Boolean variables, namely $x_{i}$. Second, they defined $E_{F}$ as $\left\{\left\{u,v\right\}|x=\phi^{-1}\left(u\right),y=\phi^{-1}\left(v\right)\mathrm{and}y=N\left(x,F\right)\right\}$, i.e., two vertices in a variable graph $G(F,W_{F})$ are connected if and only if their corresponding Boolean variables are neighbors in *F*. Third, the edge weight component $W_{\langle F,W_{F}\rangle }$, is defined as below.

W〈F,WF〉({u,v})=∑x,y∈C1|C|2,

where $x=\phi^{-1}\left(u\right),y=\phi^{-1}\left(v\right)$. In this formula, |C| is the cardinality of *C* and |C|2 means a combination of |C| elements taken two elements at a time. The motivation is to give the same relevance to all clauses, so they pondered the contribution of a clause to an edge by 1/|C|2. This way, the sum of the weights of the edges generated by a clause is always 1. In this paper, we propose an extension to this weighting scheme that is tailored for PMS and WPMS.

### 2.2. Local Search for MaxSAT

The basic framework of SLS algorithms for solving (W)PMS can be described as follows. Initially, an SLS algorithm randomly generates an assignment of Boolean values to all variables; then, it repeatedly selects and flips a Boolean variable until the cutoff arrives; finally, it returns the best feasible assignment that has been found. During the search, most SLS algorithms alternate between two modes: greedy (intensification) mode and random (diversification) mode. In greedy modes, SLS algorithms prefer those flips that lead to a decreasing number of unsatisfied hard clauses and a decreasing total weight/number of unsatisfied soft clauses. In random modes, they tend to diversify the search by randomized strategies.

### 2.3. Clause Penalties in SATLike 3.0

Although greedy search helps find better solutions nearby, it can often be trapped in local optima, so various diversification strategies have been proposed to tackle this problem including dynamic local search. Usually, in the context of SAT/MaxSAT, SLS algorithms associate each clause in a CNF formula *F* with a penalty, in order to help focus more on those clauses that are rarely satisfied [38]. To be specific, if a clause, namely *C*, is often unsatisfied, they will increase *C*’s penalty often. As a result, any solution that violates *C* will tend to have a great penalty. Alternatively, the search will have high priority to satisfy *C*. In this sense, each clause will have opportunities to be satisfied.

Below, we introduce the penalty management scheme in SATLike 3.0 [37], which is also adopted for our algorithms. It distinguishes between hard and soft clauses with three parameters: the change $\delta_{h}$ for hard clause penalties, the change $\delta_{s}$ for soft clause penalties, as well as Λ, which limits soft clause penalties. SATLike 3.0 uses Λ to prevent the penalties of soft clauses from being too large, in case they receive too much attention. Furthermore, $\delta_{h}$ is usually greater than $\delta_{s}$ because hard clauses should have greater impacts on the search, compared to soft ones:

1. Initially:
  (a) $\mathrm{penalty}(C^{h})\leftarrow 1$ for each hard clause $C^{h}$;
  (b) $\mathrm{penalty}\left(C^{s}\right)\leftarrow W_{F}\left(C^{s}\right)$ for each soft clause $C^{s}$.
2. At each local optimum:
  (a) with probability *p*:
    i. $\mathrm{penalty}\left(C^{h}\right)\leftarrow \mathrm{penalty}\left(C^{h}\right)+\delta_{h}$ for each violated hard clause $C^{h}$;
    ii. $\mathrm{penalty}\left(C^{s}\right)\leftarrow \mathrm{penalty}\left(C^{s}\right)+\delta_{s}$ for each violated soft clause $C^{s}$ s.t. $\mathrm{penalty}(C^{s})<\Lambda$.
  (b) with probability 1−p:
    i. $\mathrm{penalty}\left(C^{h}\right)\leftarrow \mathrm{penalty}\left(C^{h}\right)-\delta_{h}$ for each satisfied hard clause $C^{h}$ s.t. $\mathrm{penalty}\left(C^{h}\right)>\delta_{h}$;
    ii. $\mathrm{penalty}\left(C^{s}\right)\leftarrow w\left(C^{s}\right)-\delta_{s}$ for each satisfied hard clause $C^{s}$ s.t. $\mathrm{penalty}(C^{s})$$>\delta_{s}$.

Like most SLS algorithms, SATLike 3.0 uses the traditional notion of a *score* to select variables to flip, in order to decrease the total penalties. Before introducing this notion, we first introduce the cost of an assignment over a MaxSAT formula, which sums up the penalties of all hard and soft clauses. Given an assignment α, the *cost* of α over $\langle F,W_{F}\rangle$, denoted by $\mathrm{cost}(\alpha ,F,W_{F})$, is defined as the total penalties of all unsatisfied clauses. In this sense, the score of *x* under α over $\langle F,W_{F}\rangle$, denoted by $\mathrm{score}(x,\alpha ,F,W_{F})$, is defined as the benefits of flipping *x* in *F*. More specifically,

$$\mathrm{score}\left(x,\alpha ,F,W_{F}\right)=\mathrm{cost}\left(\alpha ,F,W_{F}\right)-\mathrm{cost}\left(\alpha^{\prime },F,W_{F}\right),$$

where $\alpha^{\prime }$ is the same as α with *x* being flipped. Therefore, the scoring function measures that decrease of penalties that is caused by flipping *x*. SATLike, as well as its variants mainly rely on this scoring function and guide local search to seek a better solution. Last, we use age(x) to denote the number of flips since the last time *x* was flipped.

### 2.4. SATLike 3.0

Below, we introduce a state-of-the-art solver, SATLike 3.0 (See Algorithm 1) [37], which performed well in recent MaxSAT Evaluations. It is named after SAT because it works somewhat like a SAT solver. The experimental results showed that it outperforms some SAT-based solvers in some industrial benchmarks.
**Algorithm 1:** SATLike 3.0.    **input**  : A (W)PMS formula $\langle F,W_{F}\rangle$ and the *cutoff*    **output**: A best solution found $\alpha^{*}$ or “NO SOLUTION FOUND”
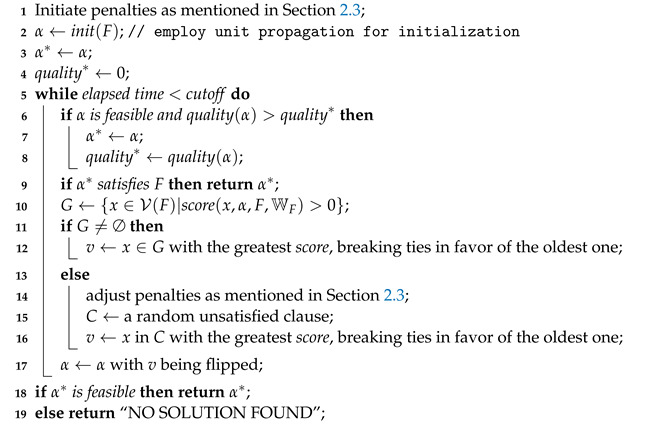


## 3. Initiating a Solution Based on Variables’ Structural Entropy

In local search, an initial solution (starting point), which lies near a high-quality one, may cost significantly less steps (flips) to achieve that satisfactory solution. In our previous works [39,40], we confirmed that a variable’s structural entropy significantly influences the probability that it will be flipped later in local search. Based on this result, we developed strategies for initiating solutions and such strategies greatly improve two state-of-the-art solvers, Sparrow and CCASat. However, most SLS PMS and WPMS solvers initiate a solution in a purely random way, which possibly generates a bad starting point. Hence, in this work, we extend our initiating strategy for SAT to (W)PMS problems, in order to improve efficiency. In this section, we introduce (1) a novel weighting scheme for the variable graph of a MaxSAT formula, (2) our definition of structural entropy, and finally, (3) our algorithm for constructing a good starting point.

### 3.1. A Novel Weighting Scheme Tailored for (W)PMS

We adopted the notion of the variable graph from [21], but assigned each edge a positive weight in a novel approach. Before introducing this approach, we first define the *relevance* of any pair of distinct variables in a MaxSAT formula.

**Definition 1.**  *Given a (weighted) partial MaxSAT formula $\langle F,W_{F}\rangle$, a clause C∈C(F), and a pair of variables x,y, we define the* relevance *between x and y in C over $\langle F,W_{F}\rangle$, denoted by $t_{\langle F,W_{F},C\rangle }\left(x,y\right)$, as below, where W is the total weight of all soft clauses.*

t〈F,WF,C〉(x,y)=1|C|2ifx,y∈CandC∈Ch(F);WF(C)W·1|C|2ifx,y∈CandC∈Cs(F);0otherwise.

Now, we discuss some special cases of this formula to present some intuition:

1. If either *x* or *y* is absent from *C*, we think that there is no connection between them in *C*, so the relevance between them in *C* is defined to be 0.
2. If *C* is long, we believe that the connection between *x* and *y* is weak, so |C|2 will be big and the relevance tends to be small. Cases are analogous if *C* is short.
3. If *C* is soft, we guess that their connection is weak, so the coefficient $\frac{W_{F}\left(C\right)}{W}$ helps decrease the relevance value.
4. If *C* is a soft clause with a great weight, we feel that the connection between *x* and *y* is relatively big, then $\frac{W_{F}\left(C\right)}{W}$ will be relatively big as well and so will be the relevance.

Then, we define the total relevance between *x* and *y* in a clause set *S* over $\langle F,W_{F}\rangle$ as the sum of relevance over all clauses in *S*, as is shown below.

$$t_{\langle F,W_{F},S\rangle }\left(x,y\right)=\sum_{C\in S}^{}t_{\langle F,W_{F},C\rangle }\left(x,y\right)$$

Here, we abuse the notation in Definition 1 and write $t_{\langle F,W_{F},S\rangle }\left(x,y\right)$ to discuss cases about clause sets. Therefore, the total relevance between *x* and *y* in the clause set of *F*, i.e., C(F), is $t_{\langle F,W_{F},C\left(F\right)\rangle }\left(x,y\right)$, which measures how closely related the two variables are in the involved MaxSAT formula.

Finally, we are ready to define edge weights in $G(F,W_{F})$, i.e., $W_{\langle F,W_{F}\rangle }\left(u,v\right)=t_{\langle F,W_{F},C\left(F\right)\rangle }\left(x,y\right)$, where $x=\phi^{-1}\left(u\right)$ and $y=\phi^{-1}\left(v\right)$. In this sense, the weight of an edge in our variable graph $G(F,W_{F})$ represents the relevance between their corresponding Boolean variables in the MaxSAT formula $\langle F,W_{F}\rangle$.

### 3.2. Properties of Our Weighting Scheme

Now, we discuss the impacts of hard and soft clauses on the relevance between Boolean variables. First, we have a proposition below that shows that the contribution of a *single* hard binary clause to the relevance is no *smaller* than that made by all soft clauses.

**Proposition 1.**  *Given a MaxSAT formula $\langle F,W_{F}\rangle$, if there exists a binary hard clause $C^{h}$ that contains variables x and y, then*

$$t_{\langle F,W_{F},C_{s}\left(F\right)\rangle }\left(x,y\right)\leq t_{\langle F,W_{F},C^{h}\rangle }\left(x,y\right);$$

*the equality relation holds if and only if all soft clauses are of length 2.*

**Proof.**  First, we amplify the left-hand side as below.

(1) t〈F,WF,Cs(F)〉(x,y)=∑C∈Cs(F)t〈F,WF,C〉(x,y)=∑C∈Cs(F),|C|≥2WF(C)W·1|C|2≤∑C∈Cs(F),|C|≥2WF(C)W·1|Ch|2=1|Ch|2·∑C∈Cs(F),|C|≥2WF(C)W

(2) ≤1|Ch|2=t〈F,WF,Ch〉(x,y).

1. As to (1), if there exists any soft clause whose length is greater than 2, then the equation there does not hold.
2. As to (2), if there exists any soft clause whose length is smaller than 2, then the equation there does not hold.
3. Obviously, if all soft clauses are of length 2, the equality relation in the proposition above holds.

According to the statements above, we have proven this proposition.    □

Based on this proof, we have a corollary below.

**Corollary 1.**  *Given a MaxSAT formula $\langle F,W_{F}\rangle$ and an integer k s.t. k≥2, if:*

*1*. *there exists a hard clause $C^{h}$ of length k that contains variables x and y;*
*2*. *all soft clauses are at least of length k.*

*then*

$$t_{\langle F,W_{F},C_{s}\left(F\right)\rangle }\left(x,y\right)\leq t_{\langle F,W_{F},C^{h}\rangle }\left(x,y\right);$$

*the equality relation holds if and only if all soft clauses are of length k.*

### 3.3. Variables’ Structural Entropies

Given an edge-weighted graph $G=(V,E,w_{G})$, we use N(u,G) to denote the set of *u*’s neighbor in *G* and use d(u,G) to denote the cardinality of N(u,G), i.e., d(u,G)=|N(u,G)|. Moreover, we use ω(u,G) to denote *u*’s weighted degree, i.e., $\omega \left(u,G\right)=\sum_{v\in N(u,G)}w_{G}\left(\left\{u,v\right\}\right)$, suppressing *G* if understood from the context. Given U⊆V, we define the volume of *U*, denoted by vol(U), as $\sum_{u\in U}\omega \left(u\right)$, and we abuse this notation to define vol(G) as vol(V).

**Definition 2.**  *Given a (weighted) partial MaxSAT formula $\langle F,W_{F}\rangle$ and its variable graph $G\left(F,W_{F}\right)=\left(V_{F},E_{F},W_{\langle F,W_{F}\rangle }\right)$, where $V_{F}=\left\{v_{1},\cdots ,v_{n}\right\}$, we define $v_{i}$’s structural entropy as*

$$H\left(v_{i}\right)=-p_{i}\log_{2}p_{i}=-\frac{\omega (v_{i})}{\mathrm{vol}(G)}\log_{2}\frac{\omega (v_{i})}{\mathrm{vol}(G)},$$

*then the structural entropy of the variable graph G is defined as*

$$H\left(G\right)=\sum_{i=1}^{n}H\left(v_{i}\right).$$

As is stated in [41], the structural information H(G) of a weighted and connected graph *G* measures the information required to determine the code of the vertices that are accessible from a random walk in *G* with its stationary distribution $\left(p_{1},\cdots ,p_{n}\right)$. On the other hand, as to a single vertex, namely $v_{i}$, $H(v_{i})$ represents the uncertainty information of a random walk with a stationary distribution to visiting $v_{i}$ from its neighbors.

Then, given a MaxSAT formula $\langle F,W_{F}\rangle$ and a variable, namely *x*, we abuse the notation above to define *x*’s structural entropy in $\langle F,W_{F}\rangle$ as the structural entropy of its corresponding vertex in $G(F,W_{F})$.

Now, we present some properties of our definition of structural entropy to help understand its insights intuitively.

**Proposition 2.**  *Let $f\left(x\right)=-x\log_{2}x$ with x∈(0,1); we have:*

*1*  *f(x)>0 for any x∈(0,1);*
*2.*  *f(x) is strictly monotonically increasing (respectively decreasing) in (0,1/e) (respectively (1/e,0)), where e is Euler’s constant and e≈2.71828⋯.*

Therefore, given a vertex, namely $v_{i}$, with $H\left(v_{i}\right)=-p_{i}\log_{2}p_{i}$, if $H(v_{i})$ is relatively small, then $p_{i}$ is relatively close to 0 or 1. Similarly, if $H(v_{i})$ is relatively large, then the value of $p_{i}$ is near 1/e. In our algorithm, we first assign Boolean variables whose corresponding vertex has relatively small structural entropy. Now, we explain the motivation as follows. Vertices with relatively small structural entropy correspond to Boolean variables that are of *much* or *little* influence on other variables in the CNF formula. Below, we discuss these cases in details:

1. Assigning highly influential variables tend to satisfy relatively many clauses or help satisfy clauses with great weights.
2. Variables of little influence often occur in few clauses or in clauses of small weights, so we simply assign them to satisfy such clauses.

### 3.4. Initiating Solutions

In this subsection, given a CNF formula *F*, we use C(F,x) to denote the set of clauses in *F* (including both hard and soft ones) that contain *x* as one of its literals. Similarly, we define the notation of C(F,¬x). Then, our procedure for initiating a solution is described in Algorithm 2, which is named variables’ structural entropy-based initialization (VSEI). The motivation of VSEI is as follows. The smaller a variable’s structural entropy is, the more stable its truth value is, hence the smaller the probability that it will be flipped later [40]. That is, a variable with smaller structural entropy should probably be assigned earlier, compared to those variables with greater ones.

The main idea of Algorithm 2 is as follows. When initiating a solution, we repeated the following operations: picking a variable that is unassigned with the smallest structural entropy and, then, mapping it to 0 or 1, depending on the number of its positive and negative occurrences in clauses that have not been satisfied yet. To be specific, suppose we have picked a variable *x* and *x*’s positive occurrences is more than its negative ones, then we assign 1 to *x*; otherwise, we assign 0 to *x*. In a nutshell, we assign values to variables greedily in order to satisfy as many clauses as possible at the end of initialization.
**Algorithm 2:** VSEI.    **input**  : A (W)PMS formula $\langle F,W_{F}\rangle$    **output**: An initial solution
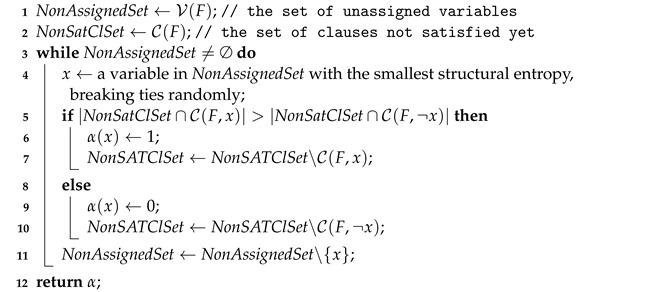


## 4. Diversifying Variable Selection Based on Clause Penalties

Each time the search encounters a local optimum, i.e., there are no variables whose flip leads to a penalty decrease, we will call Algorithm 3 to pick a variable and flip it. More specifically, this algorithm first adjusts penalties like SATLike 3.0 (Line 1), then it picks a variable in two possible ways depending on a parameter *p*: (1) continuing to choose one with the best score and the best age (Line 3); (2) focusing on an unsatisfied clause with the greatest penalty (Line 5) and performing probabilistic selections on it (Line 6). Considering its most distinguishing features, we name it probabilistic selection for great penalties (PSGP).
**Algorithm 3:** PSGP.    **input**  : score(x) and age(x) for all *x*’s in *F*, the current unsatisfied clause set *U*    **output**: A variable to be flipped
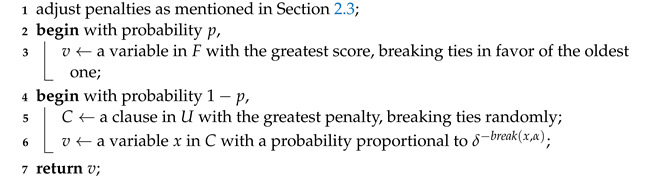


In Line 3, we insist on picking the globally best variables, and this may bring some benefits. The reason is that Line 1 just changes the penalties of some clauses, which, in turn, affects the score of some variables involved.

In Line 6, each variable namely *x* in *C* is picked with a probability proportional to $\delta^{-\mathrm{break}(x,\alpha )}$, where break(x,α) is the number of clauses (including both hard and soft ones) that will become unsatisfied if *x* is flipped, given the current assignment α. Hence, this probability distribution always prefers small-break variables. Here, the parameter δ controls how concentrated this distribution is at small-break variables. Obviously, the greater δ is, the greater the probability difference between small-break and big-break variables. This distribution is inherited from ProbSAT [42], which was a simple and elegant local search SAT solver with a probabilistic selection as its single strategy.

## 5. ImSATLike

In this section, we introduce our novel Algorithm 4 as a whole, which works on (W)PMS instances. In the initialization procedure, it adopts variables’ structural entropy to generate a good starting point of local search. Then, during local search, each time it meets a local optimum, it will still pick the globally best variables or it will focus on an unsatisfied clause with the greatest penalty and choose variables by probabilistic selection. Since our algorithm is based on SATLike 3.0, we call it ImSATLike.

There are two main differences between our algorithm and SATLike 3.0: (1) SATLike 3.0 employs unit propagation to generate an initial solution, while our solver initiates starting points by variables’ structural entropy; (2) in diversification, SATLike 3.0 picks a random unsatisfied clause and performs greedy selection, while our solver still possibly continues our greedy strategy or focuses on a clause with the greatest penalty and exploits probabilistic selection.
**Algorithm 4:** ImSATLike.    **input**  : A (W)PMS formula $\langle F,W_{F}\rangle$ and the *cutoff*    **output**: A best solution found $\alpha^{*}$ or “NO SOLUTION FOUND”
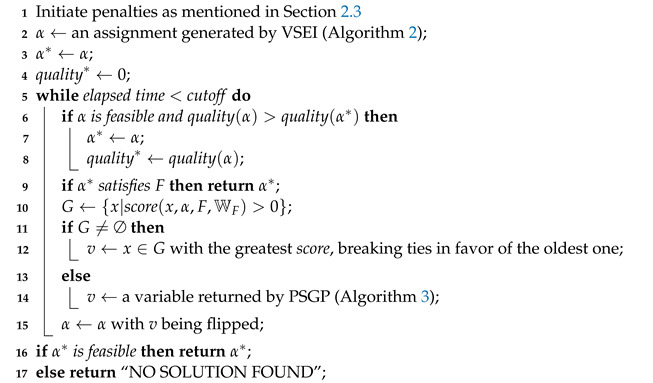


## 6. Experimental Evaluations

To evaluate the performance of our algorithm, we compared it to SATLike and its improvement SATLike 3.0 on (W)PMS instances, which was used in MaxSAT Evaluations 2018 and 2019. To be specific, these instances came from four benchmarks, namely ms18_wt, ms19_wt, ms18_unwt, and ms19_unwt, among which the former (respectively latter) two contain all weighted (respectively unweighted) partial MaxSAT instances used in 2018 and 2019, respectively. For each instance, namely I, among I’s feasible solutions, there is a quality that is known to be the best, and we call it I’s best-known (solution) quality. Given a solver A and an instance I, we say that A*successfully* solves I in a particular run if A locates a solution of that best-known quality in that run.

SATLike 3.0 not only outperforms CCEHC and Dist, but also beats their respective improvements, DeciDist and DeciCCEHC, which alternates between decimation and local search [43]. Hence, it is the current best local search solver for (W)PMS problems. In addition, we also compared our solver to two SAT-based ones, Open-WBO-inc [30] and LinSBPS (https://helda.helsinki.fi/bitstream/handle/10138/237139/mse18_proceedings.pdf?sequence=1, accessed on 8 November 2022), which were the top two solvers in MaxSAT Evaluation 2018.

SATLike 3.0 was downloaded from the web pages of MaxSAT Evaluation 2018 (https://maxsat-evaluations.github.io/2018/, accessed on 8 November 2022), and we adopted its default parameter settings in the following experiments. Based on this version, we developed ImSATLike with two extra parameters: *p* and δ in Algorithm 3, which were set to 0.3 and 2.06, respectively.

In the following tables, we use ins_class to denote instance sets, #win to denote the number of instances that were successfully solved, #ins to denote the number of instances in each instance set, and time to denote the average running time to locate a solution. The best #win and time values are shown in **bold** font.

### 6.1. Comparing ImSATLike to Other SLS Solvers

We compared ImSATLike with SATLike, as well as its 3.0 version on a computer equipped with an Intel(R) Core(TM) i5-10210U CPU @ 1.60 GHz 2.11 GHz with 8 GB RAM, running the Windows 10 OS. First, we conducted experiments with 60 s as a cutoff, then we repeated such experiments with 300 s as a second cutoff (see Table 1).

From Table 1, we found the following. Within 60 s:

1. ImSATLike outperformed both SATLike and its 3.0 version in terms of #win on each of the four benchmark categories, with the exception of ms18_unwt, where ImSATLike and SATLike came to a draw;
2. In this benchmark category, ImSATLike generally located its solutions within a shorter time, compared to that spent by SATLike.

Within 300 s:

1. ImSATLike showed best performances in terms of #win in 3 categories, while SATLike did that in 2;
2. In ms19_wt, where both ImSATLike and SATLike achieved 162 in terms of #win, ImSATLike generally located its solutions within a much shorter time, compared to that spent by SATLike.

### 6.2. Individual Impacts of Our Strategies

To evaluate the individual impacts, we modified SATLike 3.0 and developed two *independent* variants. Then, we reperformed the experiments above with 60 s as the cutoff and compared these variants in terms of #win.

1. First, we replaced the initialization procedure in SATLike 3.0 with our VESI strategy and developed a solver named SATLike_a1.
2. Second, we replaced SATLike 3.0’s diversification mode with our PSGP strategy and developed a second solver named SATLike_a2.

From Table 2, we found the following:

1. In none of the benchmark categories, SATLike 3.0 outperformed either SATLike_a1 or SATLike_a2 in terms of #win, which illustrates the robustness of our strategies.
2. In all of these categories, SATLike_a2 significantly outperformed SATLike 3.0, which showed the power of our PSGP strategy.
3. In half of these categories, SATLike_a1 was superior to SATLike 3.0, which presented the positive impacts of our VESI strategy.

### 6.3. Comparing ImSATLike to SAT-Based Solvers

We compared ImSATLike with two SAT-based solvers, Open-WBO-Inc and LinSBPS, on a computer equipped with an Intel Core i5-10210U CPU @ 1.60 GHz × 8 with 8 GB RAM, running Ubuntu 18.04.5 LTS. These two competitors were the top two solvers in the incomplete track in MaxSAT Evaluation 2018, where the time limit was 60 s, and their codes were downloaded from the web pages of MaxSAT Evaluation (https://maxsat-evaluations.github.io/2018/, accessed on 8 November 2022). To be consistent with MaxSAT Evaluation 2018, we also set the cutoff here to be 60 s. From Table 3, we found that our solver performed somewhat close to the top SAT-based solvers on weighted partial MaxSAT instances, although it fell behind in general.

### 6.4. Evaluations of a Hybrid Solver Incorporating ImSATLike

Combining solvers in different frameworks has proven to be a promising approach, which has been confirmed in recent MaxSAT Evaluations. Therefore, we combined our solver ImSATLike with a state-of-the-art SAT-based solver, TT-Open-WBO-inc [31], which was the champion in the incomplete track of MaxSAT Evaluation 2019. We call this hybrid solver ImSATLike-TT, and its work flow based on a MaxSAT formula is as follows:

1. A SAT solver B is called to find a feasible solution $\alpha_{\mathrm{init}}$.
2. B passes $\alpha_{\mathrm{init}}$ to ImSATLike as its starting point.
3. ImSATLike bypasses its VSEI strategy and performs local search for *k* steps, where *k* was set to be $10^{7}$.
4. ImSATLike passes its best-found solution to TT-Open-WBO-inc.
5. TT-Open-WBO-inc is run for the remaining time.

In most cases, ImSATLike was able to find high-quality solutions, but the time for it to find even better solutions will increase exponentially, so in this situation, ImSATLike-TT will turn to TT-Open-WBO-inc for better solutions.

For better comparisons, we also included TT-Open-WBO-inc and SATLike-ck as competitors. Note that SATLike-ck is the same as ImSATLike-TT, but employs SATLike as its embedded local search component. The experiments were conducted on a computer equipped with an Intel Core i5-10210U CPU @ 1.60 GHz × 8 with 8 GB RAM running Ubuntu 18.04.5 LTS, and the cutoff was set to 60 s. The experimental outcome can be found in Table 4, which shows the number of successfully solved instances for each SAT-based solver and each portfolio in each benchmark category.

In Table 4, we find the following:

1. On partial MaxSAT instances, ImSATLike-TT performed the same as SATLike-ck, and they were the top two solvers, which showed the superiority of portfolios over SAT-based solvers.
2. On weighted partial MaxSAT instances, ImSATLike-TT performed as well as SATLike-ck in ms18_wt, but outperformed it in ms19_wt, which showed the positive effects of our strategies.

## 7. Conclusions and the Future Work

In this paper, we presented a local search MaxSAT solver named ImSATLike, as well as a hybrid solver named ImSATLike-TT, which performed better than or the same as state-of-the-art competitors SATLike 3.0 and SATLike-ck, respectively, on (weighted) partial MaxSAT instances in recent MaxSAT Evaluations.

The main contributions include: (1) an initialization strategy to help generate a solution that is closer to high-quality feasible ones; (2) a diversification strategy to guide local search to a more promising area.

As for future works, it will be interesting to apply these strategies to solve other combinatorial problems such as the vertex cover and dominating set problems.

## Figures and Tables

**Table 1:** Comparative results of ImSATLike and SATLike with its 3.0 version.

ins_class	#ins	SATLike		SATLike 3.0		ImSATLike	
		#win	#time	#win	#time	#win	#time
60 s							
ms18_wt	172	101	23.2335	99	13.8662	**103**	17.4195
ms19_wt	282	154	23.4689	152	24.5351	**159**	19.5359
ms18_unwt	153	**77**	52.1586	73	50.2791	**77**	**49.0867**
ms19_unwt	288	158	14.0828	149	15.9554	**163**	16.2713
300 s							
ms18_wt	172	**110**	103.554	102	99.4858	106	76.8005
ms19_wt	282	**162**	119.761	160	91.7588	**162**	**79.0564**
ms18_unwt	153	77	88.226	73	115.1743	**86**	137.7936
ms19_unwt	288	166	68.926	152	77.1327	**168**	71.8434

**Table 2:** Individual impacts of the VSEI and PSGP strategies.

ins_class	#ins	SATLike_a1		SATLike 3.0		SATLike_a2	
		#win	#time	#win	#time	#win	#time
ms18_wt	172	99	14.603	99	13.8662	**103**	15.9523
ms19_wt	282	155	16.345	152	24.5351	**158**	26.3373
ms18_unwt	153	73	52.888	73	50.2791	**77**	52.7514
ms19_unwt	288	150	16.107	149	15.9554	**155**	16.3538

**Table 3:** Comparative results of our algorithm and two SAT-based solvers.

ins_class	#ins	ImSATLike	LinSBPS	Open-WBO-Inc
ms18_wt	172	130	164	164
ms19_wt	282	185	269	266
ms18_unwt	153	78	135	134
ms19_unwt	288	174	270	260

**Table 4:** Comparative results of SAT-based solvers and two portfolios.

ins_class	#ins	ImSATLike	LinSBPS	Open-wbo	TT-Open-WBO-inc	SATLike-ck	ImSATLike-TT
ms18_wt	172	130	**164**	**164**	161	158	158
ms19_wt	282	185	**269**	266	265	261	263
ms18_unwt	153	78	135	134	135	**144**	**144**
ms19_unwt	288	174	270	260	263	**277**	**277**

## Data Availability

The benchmarks used in this paper are available from https://maxsat-evaluations.github.io/2018/ and https://maxsat-evaluations.github.io/2019/ (accessed on 8 November 2022).
